# Evaluating Fibroblast Growth Factor 21 (FGF21) Levels Post-Gastric Sleeve Surgery in Obese Patients

**DOI:** 10.7759/cureus.66122

**Published:** 2024-08-04

**Authors:** Khalid A Al-Regaiey, Muhammad Iqbal, Mohammed A Alzaid, Osama A Alkaoud, Mohammed A Alhadyani, Osama A Alagel, Salem S Alshehri, Ibraheem Altamimi, Saud M Alsofayan

**Affiliations:** 1 Physiology, King Saud University, Riyadh, SAU; 2 Medicine, King Saud University, Riyadh, SAU

**Keywords:** fgf21, growth hormone, insulin sensitivity, gastric sleeve surgery, obesity

## Abstract

Background and objectives

Obesity is a major global health concern linked with increased risk of chronic diseases. This study aimed to assess the levels of fibroblast growth factor 21 (FGF21) in subjects with obesity after gastric sleeve surgery and explore its correlation with lipid and glycemic parameters.

Methods

This retrospective cohort study included 28 obese male subjects aged 25 to 50 years, undergoing gastric sleeve surgery. Plasma levels of FGF21 were measured by enzyme-linked immunosorbent assay (ELISA) before and six to 12 months after surgery. Other parameters including body mass index (BMI), fasting glucose, lipid profile, and insulin were also assessed and homeostatic model assessment (HOMA) was used to estimate insulin resistance.

Results

There was a significant increase in systemic FGF21 levels after surgery (45.12 vs. 126.16 pg/mL, p = 0.007). There was also a notable reduction in BMI (51.55 vs. 39.14, p < 0.001), insulin levels (20.06 vs. 8.85 mIU/L, p < 0.001), HOMA scores (6.94 to 2.49, p < 0.001), and glucose levels (7.33 vs. 6.08, p = 0.039). Lipid profile analysis post-surgery showed an increase in total cholesterol (4.38 vs. 5.09 mmol/L, p < 0.001) and high-density lipoprotein (HDL) (0.88 vs. 1.52 mmol/L, p < 0.001), with a decrease in triglycerides (1.75 vs. 1.01 mmol/L, p = 0.007). FGF21 positively correlated with growth hormone (GH), p = 0.0015, r = 0.59, and with insulin like growth factor 1 (IGF-1), p = 0.03, r = 0.431.

Conclusion

FGF21 levels were increased following gastric sleeve surgery in obese male patients and were positively correlated with growth hormone and insulin IGF-1. These findings provide insights into the metabolic alterations following bariatric surgery and highlight the potential role of FGF21 as an important molecule in obesity management and treatment.

## Introduction

Obesity continues to be a major global health issue. Recent data shows that the prevalence of obesity in adults has increased to 13% worldwide. The World Health Organization (WHO) estimates that in 2016, over 650 million adults were obese, with an additional 1.9 billion considered overweight [1,2]. This condition is closely linked with a higher risk of numerous chronic diseases, such as hypertension, cardiovascular disease, and type 2 diabetes mellitus (T2DM). Obesity is essentially characterized by abnormal or excessive fat accumulation that negatively impacts health [3,4].

Fibroblast growth factor 21 (FGF21), is a novel metabolic hormone produced primarily in the liver, adipose tissue, and pancreas [5]. Its production in the liver is particularly responsive to dietary changes [6]. FGF21 plays a crucial role in regulating carbohydrate and lipid metabolism, energy balance, and body weight. Increased circulating FGF21 levels promote weight loss, enhance energy expenditure, improve glucose homeostasis and insulin sensitivity, alleviate fatty liver disease, and stimulate fat breakdown [7-9]. Moreover, the FGF21 signaling pathway mediates ketogenic diet-induced amelioration of hepatic steatosis [10]. Plasma FGF21 levels did not significantly respond to short-term fasting but increased following short-term high-carbohydrate overfeeding [11,12]. FGF21 analogs and receptor agonists, which mimic the activity of FGF21, are emerging as potential therapeutic agents. They show promise in improving insulin sensitivity, reducing liver fat, and aiding weight loss. Their effectiveness is being explored in clinical trials for treating type 2 diabetes and metabolic dysfunction-associated steatohepatitis (MASH) [13]. Recent studies suggest that FGF21 treatment can mitigate various age-related metabolic disorders, including atherosclerosis, obesity, T2DM, and cardiovascular diseases [14,15]. Although functional studies indicate that FGF21 counteracts metabolic derangement, increased circulating levels of FGF21 were observed in subjects with obesity and metabolic syndrome [16] prompting the need for more investigations to understand the physiological role of FGF21.

Bariatric surgery is a well-established treatment option to reduce obesity and related comorbidities and improve glycemic control [17]. The metabolic effects of bariatric surgery are driven by the secretion and action of hormones involved in appetite, glucose metabolism, and energy expenditure [18-20]. However, there are other molecular mechanisms that influence the physiological effects of bariatric surgery that need to be addressed. In the current study, we aimed to investigate FGF21 plasma levels in obese male subjects before and six to 12 months after gastric sleeve surgery (GS).

## Materials and methods

Study design and setting

This retrospective cohort study was conducted in the Department of Physiology, and Obesity Research Centre, College of Medicine, King Saud University. The study subjects were 28 males with obesity aged between 25 and 50 years with a mean body mass index (BMI) of 51.55±1.91 kg/m^2^ and eligible for gastric sleeve surgery. Participants included in the study were not on any medication, had no history of renal, liver, or cardiovascular diseases, did not suffer from uncontrolled diabetes, had no severe complications after surgery, and adhered to the follow-up appointments during the first year after surgery. Informed consent was obtained from all participants, and the study was approved by the Institutional Review Board of the College of Medicine at King Saud University, Riyadh, Saudi Arabia (E-21-6147).

Sampling and data collection

Each patient underwent clinical evaluation by a physician, psychologist, and nutritionist before and at six to 12 months after surgery. Laparoscopic gastric sleeve surgery was performed by longitudinal resection of the fundus, corpus, and antrum to create a tubular duct along the lesser curvature with the preservation of the pylorus. BMI was recorded at each visit. Fasting blood samples were collected one day before and six to 12 months after surgery. Plasma was retrieved by centrifugation at 1500×g for 10 minutes, aliquoted, and stored at -80°C.

Biochemical analysis

An automated analyzer analyzed Blood samples for clinical parameters including glucose, total cholesterol, triglycerides, low-density lipoprotein (LDL), high-density lipoprotein (HDL), insulin, and HbA1c.

Plasma levels of FGF21 were measured by sandwich enzyme-linked immunosorbent assay using commercially available human FGF21 enzyme-linked immunosorbent assay (ELISA) Kit (abx250564) according to the manufacturer’s manual (Abbexa Ltd, Cambridge, UK). Briefly, patient samples (28 subjects before and after gastric sleeve surgery) and standards underwent reaction with specific antibodies coated in the microplate and incubated at 37ºC for an hour on a shaker. Following the addition of detection reagent A and detection reagent B, and washings as described 3,3',5,5' tetramethylbenzidine (TMB) substrate was added to quantify the horseradish peroxidase (HRP) enzymatic activity. The stop solution was added to stop the reaction and the optical density was measured at 450 nm by a microplate reader (EL 800, Bio Tek Instruments, USA). 

Statistical analysis

Data was analyzed using SPSS (IBM Corp. Released 2016. IBM SPSS Statistics for Windows, Version 24.0. Armonk, NY: IBM Corp). Descriptive statistics describe the categorical and quantitative variables. The mean and standard error of the mean were calculated for socio-demographic and clinical measurements before and after surgery. The Student’s t-test for paired samples was used for univariate analysis, with a significance threshold of p ≤ 0.05 and 95% confidence intervals to determine statistical significance and precision.

## Results

The study was conducted on 28 male patients with obesity with a mean age of 35.46± 1.59 (mean±SEM) years, all of whom underwent sleeve gastrectomy. The distribution of clinical characteristics is detailed in (Table 1).

**Table 1:** Comparison of clinical and biochemical parameters between baseline and at 6-12 months after surgery. BMI: body mass index; TGL: triglycerides; TC: total cholesterol; HDL: high-density lipoprotein; LDL: low-density lipoprotein; HOMA-IR: homeostasis model assessment of insulin resistance; GH: growth hormone, IGF-1: insulin like growth factor 1.

Variable	Pre-surgery Mean±SEM	Post-surgery Mean±SEM	p value
BMI (kg/m^2^)	51.55±1.91	39.14±1.67	0.001
Fasting glucose (mmol/L)	7.33±0.056	6.08±0.12	0.039
TGL (mmol/L)	1.75±0.24	1.01±0.07	0.007
TC (mmol/L)	4.38±0.15	5.09±0.15	0.001
HDL (mmol/L)	0.88±0.04	1.52±0.07	0.001
LDL (mmol/L)	2.7±0.15	2.97±0.13	0.12
Insulin (mIU/L)	20.06±2.00	8.86±1.16	0.001
HOMA-IR	6.94±1.08	2.49±0.038	0.001
GH (pg/mL)	26.35±7.05	100.36±27.25	0.02
IGF-1 (ng/mL)	4467.24±367	3829.96±267	0.09

**Figure 1:**
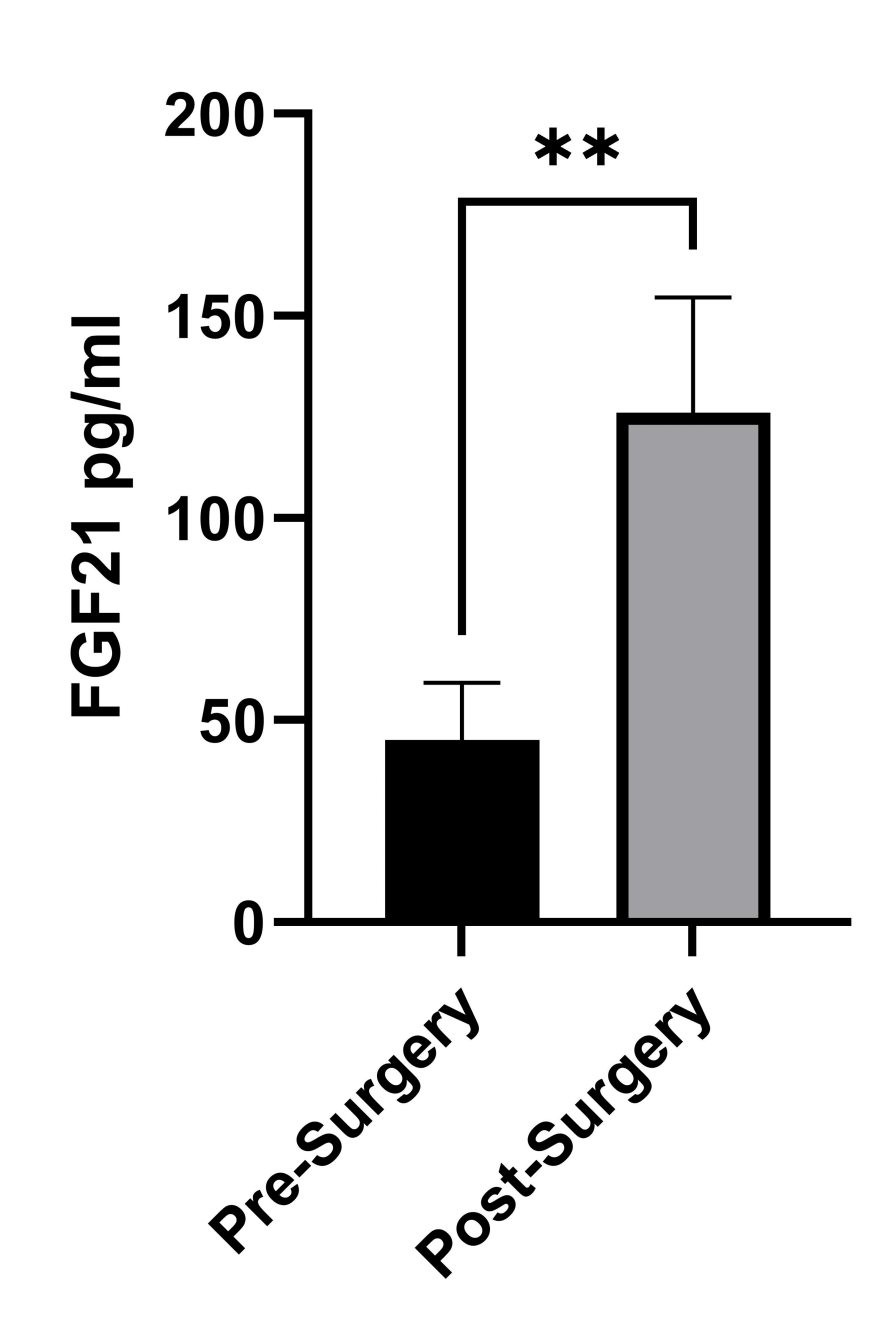
Plasma levels of FGF21 before and after gastric sleeve surgery. The error bars represent standard error of the mean. **Statistically significant differences and values of p = 0.007 indicate statistical significance.

BMI was decreased (51.55±1.91 vs. 39.14±1.67 kg/m2 [mean ± SEM], six to 12 (8.12±0.26, mean ± SEM) months after gastric sleeve surgery, p < 0.001). Fasting blood glucose was also decreased after surgery (7.33±0.056 vs. 6.08±0.12 mmol/L, p = 0.039). Total cholesterol (TC) and high-density lipoprotein (HDL) levels were increased post-surgery (4.38±0.15 vs. 5.09±0.15 mmol/L and 0.88 ± 0.04 vs. 1.52 ± 0.07 mmol/L, p < 0.001, respectively). Levels of triglycerides (TGL) were decreased after surgery (1.75 ± 0.24 vs. 1.01 ± 0.07 mmol/L, p =0.007, Table 1). Insulin levels were decreased from 20.06 ± 2.0 to 8.86 ± 1.16 mIU/L, p < 0.001, and the homeostatic model assessment-insulin resistance (HOMA-IR) index was reduced from 6.94 + 1.08 to 2.49 ± 0.04, p < 0.001, Table 1). There was no significant difference in LDL level before vs. after surgery. The mean FGF21 levels were significantly increased after surgery (45.12 ± 14.09 vs. 126.16 ± 28.47 pg/mL, p=0.007), Figure 1.

Pearson correlation analysis revealed a positive correlation between FGF21 and growth hormone (GH) after surgery, p = 0.0015, r = 0.59, and with IGF-1, p = 0.03, r = 0.431, Table 2.

**Table 2:** Correlation between FGF21 and GH and IGF-1 post-surgery. GH: growth hormone, IGF-1: insulin like growth factor 1

Parameters	r value	p value
GH	0.59	0.0015
IGF-1	0.431	0.03

## Discussion

Animal studies have shown that FGF21 has multiple beneficial effects on obesity and its related metabolic complications. Administration of FGF21 has been shown to reduce body weight, lower blood glucose and TG levels, enhance insulin sensitivity, maintain β-cell function, decrease LDL, and increase HDL [21]. In this study, FGF21 levels were analyzed in males with obesity after undergoing GS. There was a reduction in body weight, improved insulin sensitivity, reduced TG, and increased HDL along with increased FGF21 plasma levels. These findings are in line with a possible positive role for FGF21 in improving the metabolic profile after GS. 

Bariatric surgeries were previously reported to induce conflicting effects on FGF21 levels. While FGF21 plasma levels increased initially and returned back to baseline in six months in GS, Roux-en-Y gastric bypass (RYGB) on the other hand did not induce any changes [22]. In another study, both GS and RYGB induced an increase in FGF21 levels for the first three months then normalized at six months [23]. Moreover, bariatric surgery was reported to increase FGF21 levels after one month [24]. In contrast, A decrease in FGF21 concentrations in patients with obesity following weight loss achieved through diet and GS was observed, while no significant change was seen after RYGB [25]. FGF21 resistance was suggested in rodents as indicated by poor response to FGF21 administration [26]. It is, therefore, anticipated that bariatric surgery ameliorates FGF21 resistance as indicated by an improved metabolic state despite varying levels of FGF21. 

FGF21 was reported to regulate mitochondrial activity and enhance oxidative capacity in adipocytes through an AMPK-SIRT1-PGC1α-dependent pathway [27]. Moreover, metformin exerts its blood glucose-lowering effects and stimulates hepatic FGF21 expression through the adenosine monophosphate-activated protein kinase (AMPK) signaling pathway [28]. Metformin was also shown to upregulate insulin like growth factor binding protein 2 (IGFBP-2) expression through the AMPK-Sirt1-PPARα pathway [29]. Circulating FGF21 levels were increased by peroxisome proliferator-activated receptor (PPAR) α activation [30]. We previously reported that GS resulted in increased IGFBP2 [31]. These observations suggest that GS and the concomitant body weight reduction may improve insulin sensitivity in a similar way as metformin does and that FGF21 may be involved in body weight reduction and improved insulin sensitivity after GS. Furthermore, FGF21 was recently reported to mediate leptin reduction which is required for substantial weight loss in mice [32]. Although we did not measure leptin in our study, it is anticipated that FGF21 mediates leptin reduction which helps in weight loss after GS.

FGF21 is also involved in growth hormone resistance [33]. Transgenic mice overexpressing FGF21 are smaller in size than wild-type mice while FGF21-knockout mice grow bigger than wild-type mice under food-restricted conditions [34]. In our study, FGF21 levels correlated positively with growth hormone which might imply some resistance during food restrictive state of GS. IGF-1 levels in our cohort also correlated positively to a lesser extent with FGF21. FGF21 might play a role in reducing GH-induced insulin resistance after bariatric surgery.

Limitations and future directions

Our study has some limitations that warrant attention. The small sample size, exclusive inclusion of male participants, and lack of a control group may have restricted the depth of our findings. Future studies should include a more diverse demographic, particularly incorporating female subjects and control groups consisting of lean individuals or those undergoing non-bariatric laparoscopic surgery, and exploring varying follow-up time intervals post-sleeve gastrectomy, which would enhance the generalizability and depth of our findings.

## Conclusions

Our findings highlight a significant increase in FGF21 levels after gastric sleeve surgery, suggesting an important metabolic role following the procedure. FGF21 was positively correlated with GH and IGF-1. These results contribute to the growing body of evidence on the metabolic impacts of bariatric surgery and signify the necessity for further research to clarify the complex biochemical pathways involved in obesity and its treatment.
